# Frustration in Fuzzy Protein Complexes Leads to Interaction Versatility

**DOI:** 10.1021/acs.jpcb.0c11068

**Published:** 2021-03-05

**Authors:** Maria I. Freiberger, Peter G. Wolynes, Diego U. Ferreiro, Monika Fuxreiter

**Affiliations:** † Protein Physiology Lab, Departamento de Quimica Biologica, Facultad de Ciencias Exactas y Naturales, Universidad de Buenos Aires-CONICET-IQUIBICEN, Buenos Aires, 1428, Argentina; ‡ Center for Theoretical Biological Physics, Rice University, Houston, Texas 77005, United States; § Department of Biomedical Sciences, University of Padova, Padova, 35131, Italy; ∥ Laboratory of Protein Dynamics, University of Debrecen, Debrecen, 4032, Hungary

## Abstract

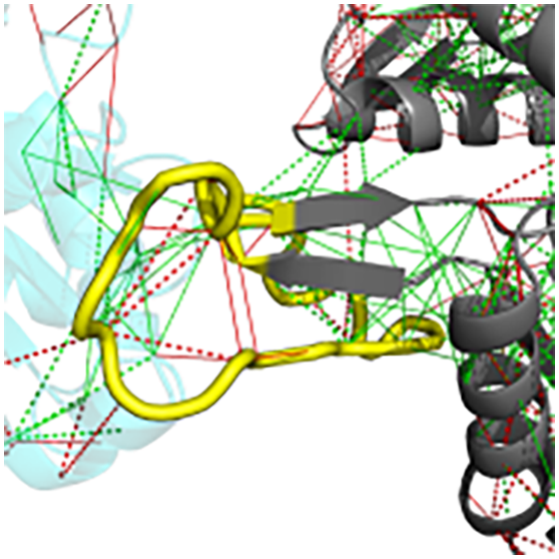

Disordered proteins frequently serve as interaction hubs involving a constrained variety of partners. Complexes with different partners frequently exhibit distinct binding modes, involving regions that remain disordered in the bound state. While the conformational properties of disordered proteins are well-characterized in their free states, less is known about the molecular mechanisms by which specificity can be achieved not with one but with multiple partners. Using the energy landscape theory concept of protein frustration, we demonstrate that complexes of disordered proteins exhibit a high degree of local frustration, especically at the binding interface. These suboptimal interactions lead to the possibility of multiple bound substates, each displaying distinct frustration patterns, which are differently populated in complexes with different partners. These results explain how specificity of disordered proteins can be achieved without a single common bound conformation and how the confliict between different interactions can be used to control the binding to multiple partners.

## Introduction

The discovery of disordered proteins has challenged the highly successful structure–function paradigm of molecular biology by raising the question of how biomolecular recognition can be achieved without a specific well-defined tertiary structure.^1,2^ Disordered proteins often function as interaction hubs in which the binding of multiple partners controls the specificity of signaling pathways.^3^ While in the past two decades a series of experimental and computational methods have begun to characterize the conformational ensembles of disordered proteins in their free states,^4^ their properties in the bound state are less well understood. Disordered proteins often display different structures when they are bound with different partners. This phenomenon is termed “fuzzy binding”.^5^ The observed binding modes of disordered proteins range from becoming nearly fully ordered to forming rather disordered states in the bound complex. The structures can also change through post-translational modification or by varying cellular conditions.^6^ Fuzzy binding enables disordered proteins to interact not with every biomolecule but specifically only with a defined set of partners. The physical basis of this controlled promiscuity has not yet been revealed.

**Figure 1:**
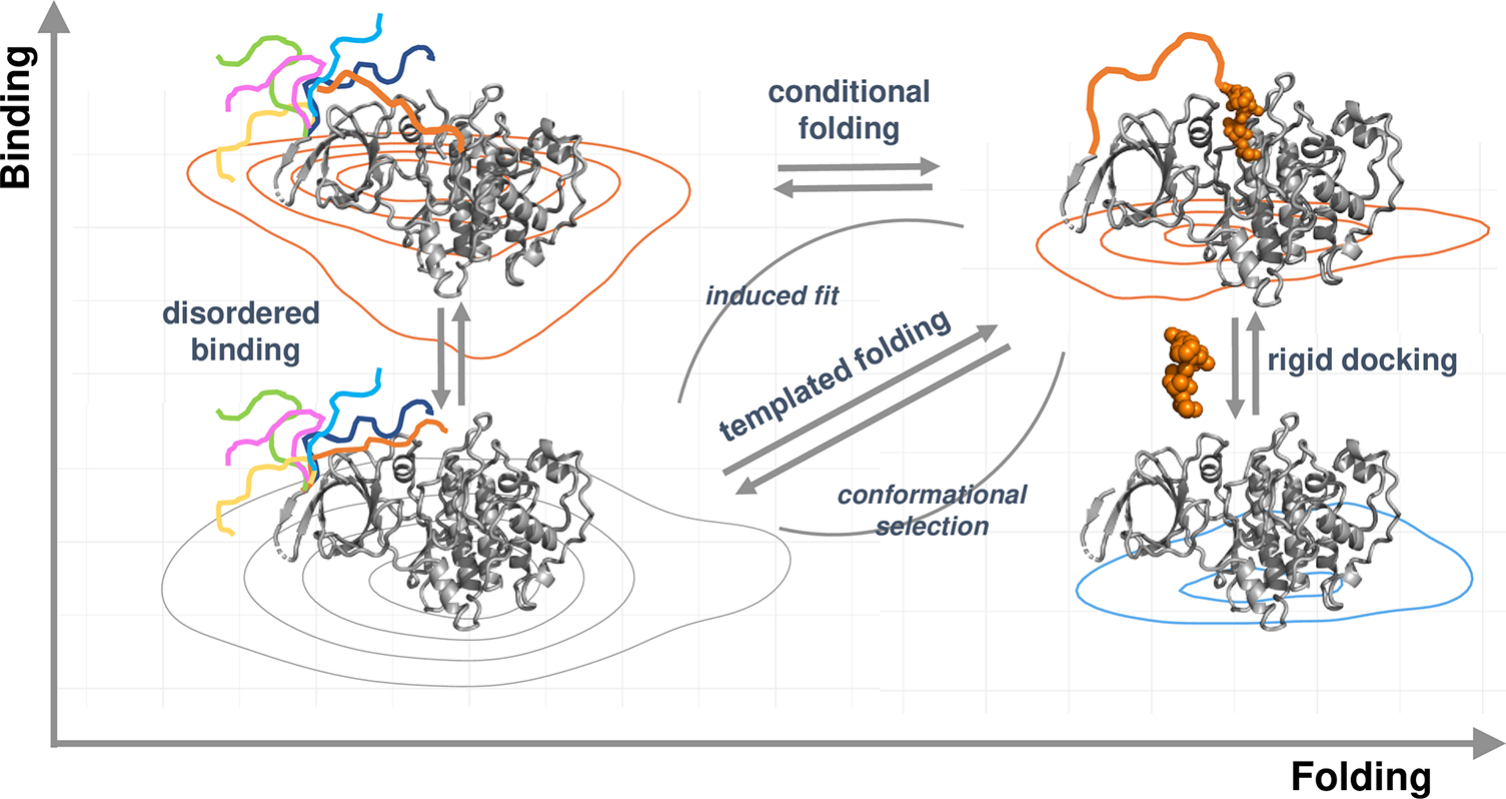
Schematic representation for folding and binding landscapes. When local frustration is low, the folded proteins associate as rigid bodies. When local frustration is high, folding is coupled to binding, templating the folding of the disordered region. If local frustration remains in the bound state, many conformations are still accessible leading to fuzziness. The structures illustrate different binding modes derived from structures of glycogen-synthase ki-nase 3 (GSK-3). The right side (vertical) represents rigid docking, when a folded protein binds to a folded partner, which in this case is the LRP6 peptide (orange) (PDB: 4nm5). The left side (vertical) represents disordered binding, when the disordered N-terminal region of GSK3 makes transient interactions with the active site. The different conformations are schematically represented by colored lines. The process displayed on top (from left to right) is the conditional folding, when the N-terminal peptide folds upon phosphorylation and binds the active site with a well-defined conformation. This structure (PDB: 4nm3) superimposes well on the complex with the LRP6 peptide (PDB: 4nm5). The orange line (top right) emphasizes that a part of the N-terminal region remains to be disordered in the complex. The diagonal (from bottom left to top right) represents the templated folding, when the disordered region adopts a well-defined structure upon binding, which can be achieved via conformational selection or induced fit. This scenario is different from conditional folding when both ordered and disordered bound states can be observed.

Disordered proteins occupy a broad range of their energy landscapes. It has been established that conformational ensembles of disordered proteins are however not fully random. They rather form secondary structure elements,^7^ with many alternative intramolecular interactions leading to numerous but somewhat structurally distinct conformational substates in the native ensemble (Figure 1). It is the entropic penalty of folding, which is a bottleneck for the folding of disordered proteins.

Disordered proteins, can undergo templated folding upon binding with their partners, leading to a more well-defined conformation in the bound state.^8^ Templated folding can be described by a funnel-like free energy landscape, which is made from both intra- and intermolecular interactions, in contrast to autonomously folding proteins, the funnel of which can be generated by intramolecular interactions alone (Figure 1). Templated folding can sometimes be described as conformational selection,^9^ when one of the conformational substates already dominant in the free state is stabilized, or may be termed induced fit, when the new conformation promoted by the partner is present only in very low concentration in the free ensemble.^10^ According to either description the intermolecular interactions of disordered proteins with their partners are thought to contrast with the rugged landscape that would arise from their intramolecular interactions alone.

Templated folding differs from autonomous folding in that often a considerable portion of the protein remains disordered in the final bound complex.^11,12^ Thus, templated folding cannot always be described by a perfectly funnel-like energy landscape. Although the structural motifs found in the bound complex often overlap with the preformed conformational elements in the unbound state,^13^ distinct conformations turn out to be sampled when the protein binds to different partners. Furthermore, mutations, which stabilize binding competent secondary structure elements in the bound form, may not always improve binding affinity.^14^ Surprisingly, in these cases mutations outside the binding region often contribute to the affinity or specificity of binding.^15^ These results suggest that heterogeneous nucleation in the templated folding pathway differs somewhat from the homogeneous nucleation of single globular proteins.^16,17^ These results also suggest that the energy landscape of the bound complex is more rugged than that for more fully folded species.

Disordered proteins can also undergo disordered binding, displaying many conformational substates in the bound forms, generated by a rugged energy landscape. Conformational exchange between these substates can be observed both within and outside the binding region.^5,18^ This pattern facilitates transient interactions at the binding interface with other functional motifs.^19^ All these observations prompt the idea that the interactions of disordered proteins can be fuzzy, and that their functional versatility exploits the diversity of many different substates.^20^ Although the biological significance of fuzziness has been established,^1,5^ understanding how diversity and specificity are reconciled requires the quantitative application of energy landscape theory. One’s intuition that interactions mediated by disordered regions must always be weak is contradicted by the existence of disordered protein complexes with high affinities.^2^

Here we examine this problem using energy landscape theory tools that analyze local frustration in proteins.^21^ These approaches were originally developed to describe how individual parts of a protein guide the folding of globular proteins toward their minimally frustrated native state.^22^ Localizing frustration has given insights into their conformational motions and into their functional adaptations that conflict with folding.^23^ In this paper we expand the theory of frustration to complexes of disordered proteins, showing consistency with the energy landscape theory. In this paper we systematically analyze frustration in the free and bound states of 160 disordered proteins that have been found to form fuzzy complexes. We find that the interactions display a high degree of frustration in both the more structured and unfolded parts of disordered proteins. We also show that while templated folding reduces the level of frustration, it does not eliminate frustration entirely, reflecting the fact that intermolecular interactions in the distinct fuzzy protein complexes are suboptimal. We find there are often distinct frustration patterns in complexes with different partners, which indicates that using suboptimal interactions provides some selectivity but also enables versatility. Our results provide a consistent physical model by which energetic frustration explains the functional versatility of fuzzy protein complexes on the basis of the energy landscape theory.

## Methods

The protocols underlying the data sets have been published, as well as the data sets themselves. Therefore, here we only provide a brief description of the methods, which are detailed in previous works.^24,25^

### Regions Representing Templated Folding (Disorder-to-Order Binding Mode, DORs, Table S1)

We collected crystal structures from the PDB that have a resolution higher than 3 Å, but that have a missing electron density for at least five residues. We excluded protein sequences with post-translational modifications or that contained nonstandard amino acids. We then collected all available bound-state structures involving the disordered region in all the complexes. We analyzed the interface residues, and selected those regions, where at least 1 residue mediated an interprotein interaction (within 4.5 A from the interface). The resulting disorder to order, DOR, from the data set contained 97 nonredundant disordered regions, which were represented in 331 complexes, in which the disordered regions folded upon binding (Table S1). For the *g*(*r*) analyses we selected a nonredundant data set of 83 complexes.

### Regions Representing Context-Dependent Binding Modes (CDRs, Table S2)

Disordered regions that were observed to be as both folded and disordered in different complex structures with distinct binding partners were defined as context-dependent regions, CDR. We assembled context-dependent regions with a minimum length of five residues in the CDR data set. This data set contained 93 nonredundant disordered regions, represented in both ordered and disordered forms in 750 complex structures (1505 chains) (Table S2). For the *g*(*r*) analyses we selected a nonredundant data set of 77 complexes.

### Data set of Fuzzy Protein Structures and Local Frustration

The protein structures for DOR and CDR class were downloaded from the Protein Data Bank (PDB, https://www.rcsb.org/) and the frustration patterns were calculated using the protein frustratometer software,^26,27^http://www.frustratometer.tk/).

## Results

We calculate the local frustration patterns using the Frustratometer server^26^ which measures the contribution of specific interactions to the creation of a funneled folding landscape. The pair distribution function shows the probability of finding a frustrated interaction at a given distance from the Cα atom of IDP. The Cα are chosen from the annotated fuzzy regions (Table S1 and Table S2). For structured regions, the Cα are chosen from random picking of a continuous segment of the same length than that of the fuzzy regions on the same protein. To quantify how the local frustration patterns correspond with fuzziness we calculated the relative pair distribution functions *g*(*r*) for the locations of various classes of contacts classified by their frustration level with respect to the Cα locations of the residues found in fuzzy regions. This allows us to see the connection of frustration to fuzziness.

### Templated Folding Leaves Highly Frustrated Interactions in Fuzzy Protein Complexes

**Figure 2:**
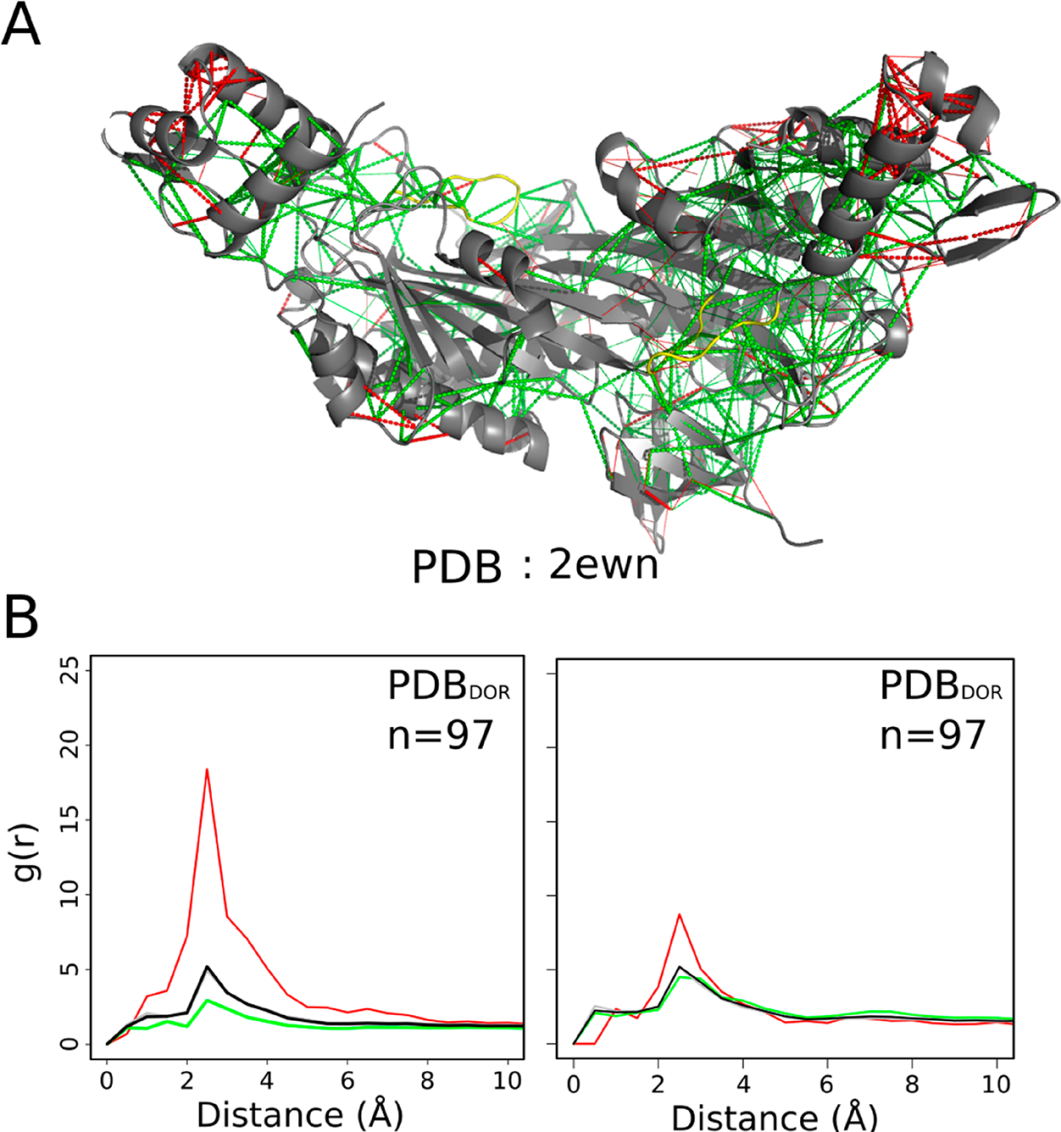
Local frustration in complexes of disordered proteins generated by templated folding where the regions undergo transition from a disordered to an ordered state. (A) Examples of frustration patterns in a protein undergoing disorder-to-order transition upon binding. The backbones of the protein are shown as gray cartoons, minimally frustrated contacts are depicted with green lines, highly frustrated interactions are depicted with red lines. Neutral interactions were omitted for clarity. The disorder-to-order region is colored yellow. (B) On the left we show the pair distribution function of the contacts between the protein and the residues in the disorder-to-order region. On the right we show the pair distribution function of the contacts between residues of structured regions: green, minimally frustrated contacts; red, highly frustrated; gray, neutral contacts; black, all contacts. In all cases *g*(*r*) values were normalized such that *g*(20) = 1.

We have evaluated the local frustration patterns in a set of 83 structures (97 fuzzy regions) of disordered protein complexes, where the disordered regions manifest disorder-to-order transitions on binding. Figure 2A shows an example of the local frustration patterns in a fuzzy protein complex. The pair distribution functions for residues classified by configurational frustration index are displayed in Figure 2B. Configurational and mutational frustration indeces differ in the way the decoys are generated. In the mutational case, the decoys are generated by sampling the change in energy given by pair-mutations on the proven contact, keeping all other interaction parameters the same. For the configurational case, the decoys are not only changed by mutations but also in distance and burial, thus sampling the distribution expected for compact non-native folds. The corresponding distributions for residues as classified by the mutational frustration index are shown in Figure S1 for all the complexes generated by templated folding. The distributions also include disordered regions which fold, but which do not mediate interactions with the partner. We found that protein regions that were originally disordered but that now adopt a well-defined structure upon binding still exhibit highly frustrated interactions between 2 and 4 Å. The density of minimally frustrated contacts is also lower in these bound but fuzzy regions (Figure 2B (left)). These results indicate that the folding of disordered regions upon binding often remains far from being optimal. We have also found that those DORs which have taken on order by templated folding, also display an enrichment in highly frustrated interactions with respect to the rest of the molecule (Figure S1A). The interactions found in the structured regions of the same proteins (chosen as random controls, Methods) also show an enrichment of highly frustrated contacts which are slightly less frustrated than those of disordered regions (Figure 2B (right)). The frustration of the interactions of the ordered regions remains significantly higher than what is usually found in complexes formed from fully structured proteins.^21^ These results indicate that templated folding of fuzzy regions also imposes constraints on the folded part of the protein.

**Figure 3:**
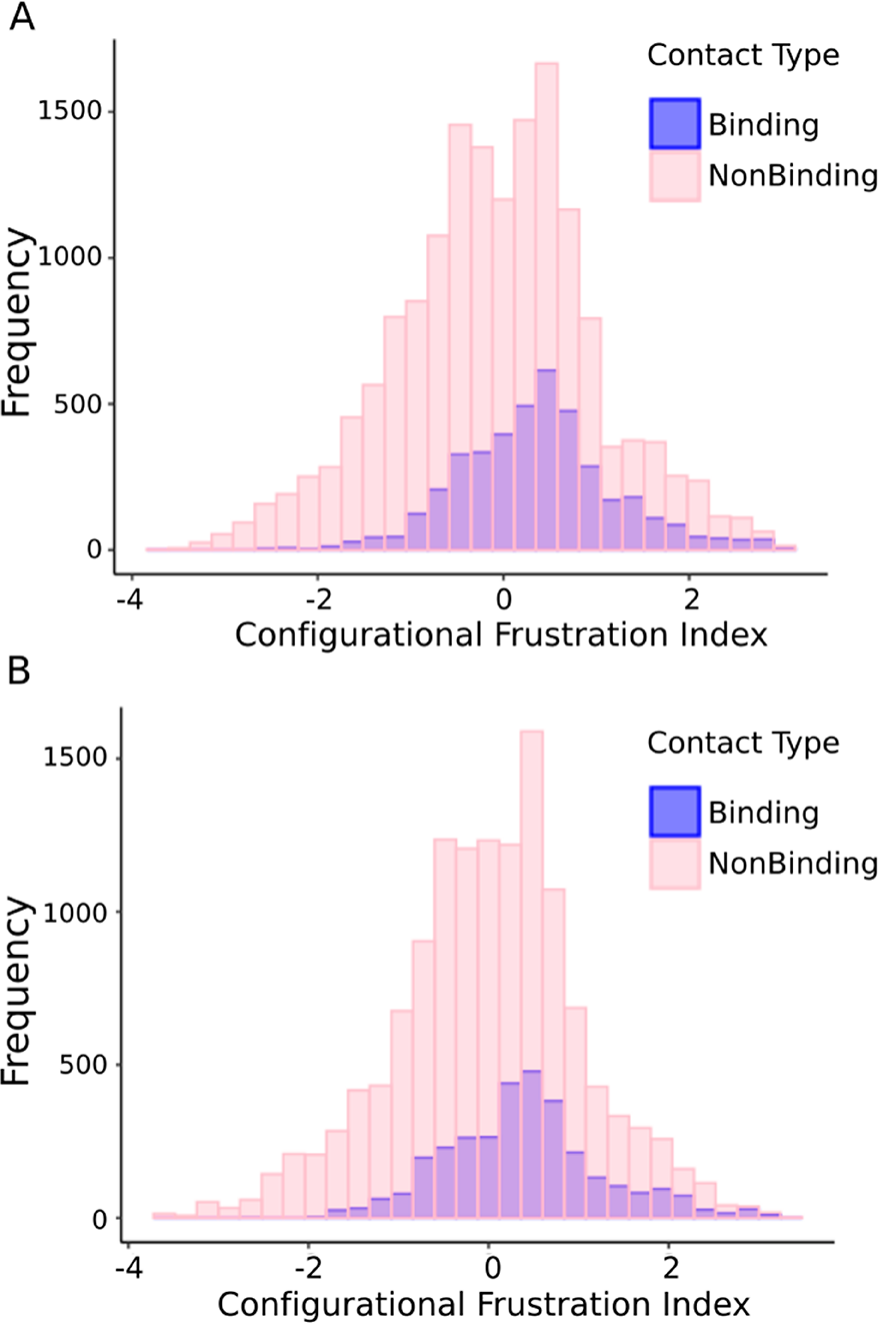
Distribution of local frustration indeces for binding contacts (red) and for the nonbinding contacts (blue) for fuzzy residues. These are shown for (A) regions representing templated folding (DOR) and for (B) regions representing context-dependent binding modes (CDR). Highly frustrated interactions are defined by the frustration index lower than −1.

We next compared the level of frustration of those residues which are involved in the binding interface (binding) to those that do not mediate intermolecular interactions (nonbinding). Highly frustrated interactions are considered if the local frustration index is lower than −1. Figure 3A compares the density of the configurational frustration index for fuzzy residues involved in binding (blue) and nonbinding contacts (pink), in the complexes generated by templated folding. We observed that those residues which do not form contacts with the partner exhibit a higher frustration index than do those which directly form intermolecular contacts (Figure 3A) indicating that the binding itself does ameliorate the high frustration of disordered proteins. These results indicate that the folding of disordered regions is less optimal than their frustrated interface interactions.

### Templated Folding Decreases the Overall Frustration of Disordered Regions Relative to the Free Monomeric State

Without simulating the intrinsically disordered protein ensemble it is difficult to assess precisely the local frustration of the disordered protein regions in their free (unbound) forms. We can get an idea of the frustration level however by examining the frustration in the structure of the disordered monomers simply by removing the interaction partner, thus generating a hypothetical single structure representative of the ensemble without intermolecular interactions. Both the finally disordered and structured regions display a higher density of frustrated contacts in the absence of the partner (Figure S2). When we compare the frustration in monomers and complexes we see that the level of frustration is lowered upon binding: partner interactions do reduce the number of suboptimal contacts (see Figure S3 and S4). For both templated folding (Figure S3) and context-dependent binding modes (Figure S4) we observe that fuzzy binding also reduces frustration as compared with the free state.

We also analyzed some monomers that are found to be ordered in the free form but become fuzzy in the bound form (Figure S5). For these 52 regions we also observe an enrichment of highly frustrated interactions around the fuzzy regions.

### Conditional Folding Is Associated with High Frustration of Disordered Regions

Increasing experimental evidence indicates that the frustrated interactions of disordered proteins^24^ often manifest themselves by forming ordered complexes with some partners but forming disordered complexes with other partners.^28^

**Figure 4:**
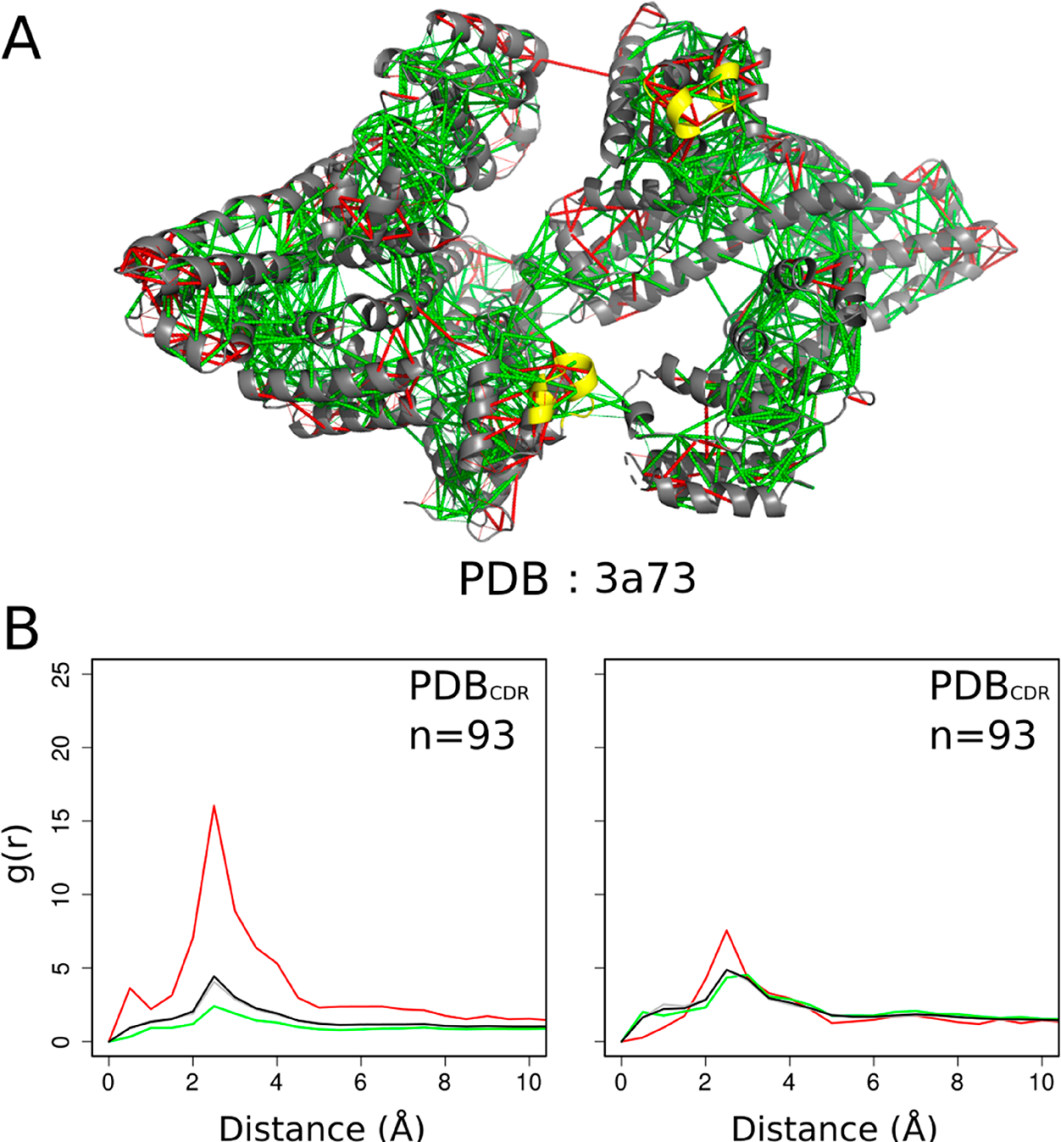
Local frustration in conditionally folding proteins. Those proteins which do not have a well-defined structure as monomer, but that may adopt a structure in a partner- or context-dependent manner or remain disordered in their complexes. (A) Examples of frustration patterns in a conditionally folding protein. The backbone of the protein is shown as gray cartoons, minimally frustrated contacts are depicted with green lines, highly frustrated interactions are depicted with red lines. Neutral interactions were omitted for clarity. The context-dependent region is marked in yellow. (B) On the left the pair distribution function of the contacts between the protein and residues of the context-dependent regions. On the right the pair distribution function of the contacts between residues of structured regions: green, minimally frustrated contacts; red, highly frustrated; gray, neutral contacts; black, all contacts; *g*(*r*) values were normalized such that *g*(20) = 1.

We term these examples as displaying “conditional folding”. In this scenario, the folding of disordered proteins depends on the binding context (context-dependent regions, CDRs), such as the interaction partner, post-translational modification or cellular conditions.^29^ Here we analyzed 77 complexes (93 fuzzy regions), generated by conditional folding and found that these complexes exhibit highly frustrated interactions (Figure 4A) similarly to templated folding (Figure 2B). The distributions for the mutational frustration indeces are shown in Figure S1. These indicate a small enrichment, relative to the topology of the protein (black lines), in frustrated interactions around the fuzzy regions. Frustrated contacts can be found both in the structured regions of the proteins (Figure 4B), and in the fuzzy regions outside the binding interface (Figure 3B). Thus, varying the degrees of folding with different partners also results in suboptimal interactions in the bound state.

### Partner-Specific Frustration Facilitates Target Selection

The above results are consistent with the idea that the folding of disordered regions upon their targets does not always result in complex structures that are completely optimal and free from conflicts. What is the molecular basis of target selection in the absence of a distinguished bound-state conformation? To answer this question, we examined some complexes in which the same disordered region interacts with different binding partners.

We illustrate the differential binding of fuzzy regions by residues 39–47 of translation initiation factor 2 subunit gamma (Q980A5). In protein structures PDB 3cw2 and PDB 3i1f eif2g folds into two different conformations. While the structure of the fuzzy region in 3cw2 is stabilized by the intramolecular interactions between Glu39–Thr46 and Glu40–Gly44, the 3i1f structure is instead stabilized by a charge–charge interaction between Glu39 and Arg43. In the 3i1f structure, the Gly44 main chain forms a hydrogen bond with a Lys42 side-chain while in the 3cw2 structure Lys42 interacts with Asp-283 of the structured domain.

**Figure 5:**
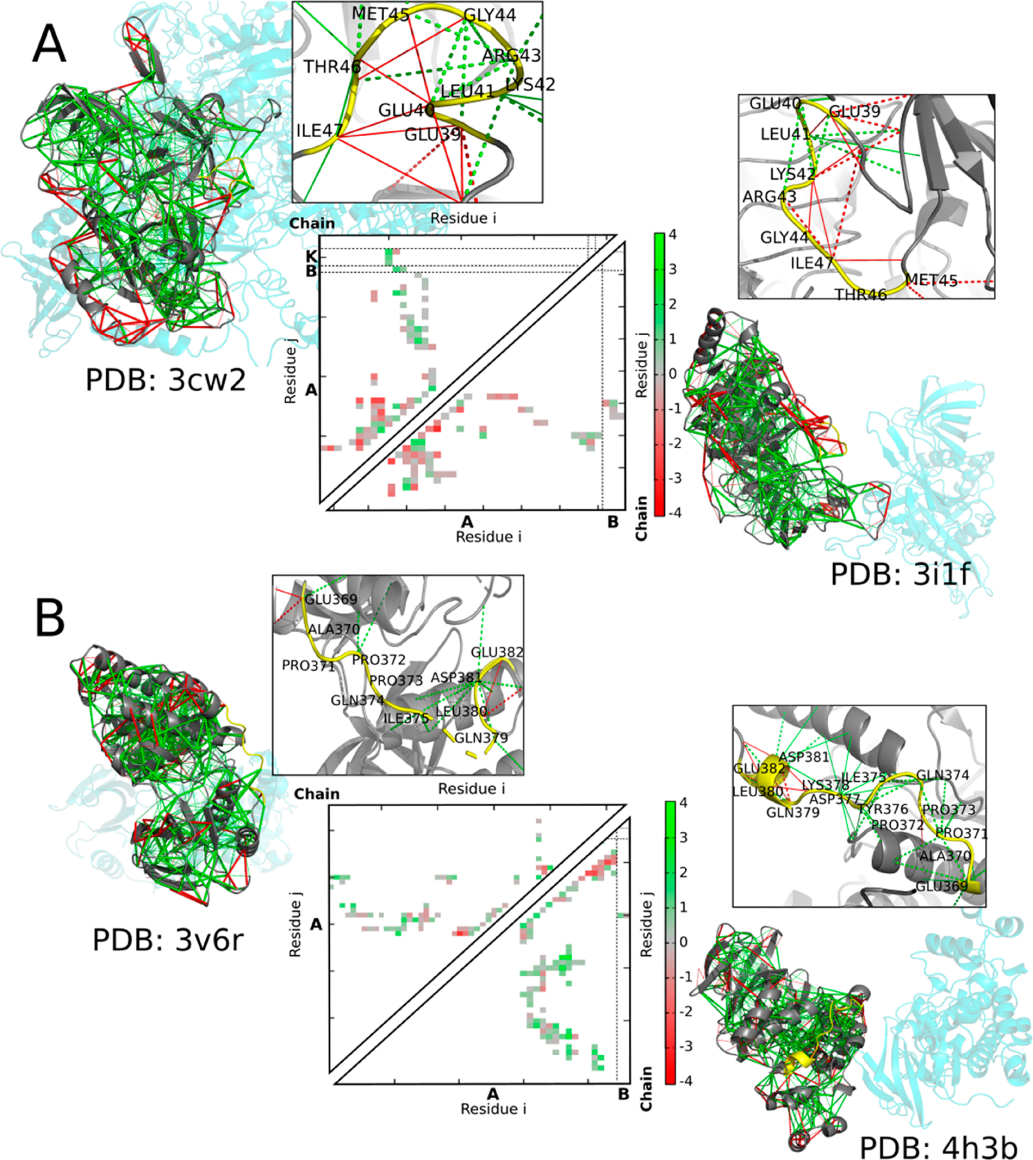
(A) Structures for translation initiation factor 2 subunit gamma (eif2g), PDB 3cw2 (above) and PDB 3i1f (below). Contact map of 3cw2 (above the diagonal), and 3i1f (below the diagonal). (B) Protein structure for mitogen-activated protein kinase 10, PDB 3v6r (above) and PDB 4h3b (below). Contact map of 3v6r (above the diagonal), and 4h3b (below the diagonal). The local frustration patterns of the protein, with the minimally frustrated interactions shown in green, the neutral shown in gray, and highly frustrated interactions shown in red. The fuzzy region is shown with the yellow backbone. For the contact map: green, minimally frustrated contacts; red, highly frustrated; gray, neutral contacts.

Figure 5A shows the structures and local frustration patterns for these different protein structures. Overall both structures possess an extended network of minimally frustrated interactions, with patches of highly frustrated interactions on the surface. Each structures display different frustration patterns for the fuzzy region in the complexes. We see that different ways of resolving the conflicts have been chosen in the alternative structures. This interchange of frustrated interactions is easily visualized in the contact maps.

Another example (Figure 5B), that illustrates the nature of fuzzy binding is the 369–382 residue region of Mitogen-activated protein kinase 10 (P53779). In the PDB 4h3b structure, the folding of the fuzzy region is stabilized by many intramolecular interactions. As we can observe in the contact map, some of these interactions form between side-chains with main-chain atoms, for example, Gln374 and Pro372, Gln379 and Leu380, or Glu382 NE2 and Glu382 C–O. In contrast, in the PDB 3v6r structure, there are considerably fewer intramolecular contacts (Glu369, Leu380, and Asp381). The contact maps clearly reflect the different ways of trying to eliminate frustration in the two complexes.

## Discussion

Some frustration in the energy landscapes is required for the functional adaptability of proteins.^23^ As a consequence of those frustrated interactions, the energy landscape is rugged encompassing distinct local minima enabling multiple biological activities.^30^ To control their interactions, generally proteins have evolved to optimize frustration allowing specificities to be compromised for functional needs.^31^ This is brought to the extreme for disordered proteins, which even lack a well-defined conformation on their own and must always be described as an ensemble of conformers.^3^ The structured character of complexes of disordered proteins with their specific partners, however, has led to the misleading impression that, in the end, functioning always requires a single well-defined conformation. The presence of a well-defined structure, however, does not correlate with the affinity of the interactions. Here we have performed a systematic analysis of the complexes of many disordered protein regions. This analysis demonstrates that even after binding, the interaction energetics are far from optimal in the fuzzy regions, in accord with experimental data.^16^ Consistent with earlier results for individual cases,^32^ we have found that both the disordered and structured regions of complexes are enriched in highly frustrated interactions in the bound complexes of disordered proteins. The interface contacts do decrease the frustration level in the disordered protein once bound as compared to the frustration of the free state, but the interactions often still remain suboptimal and energetic conflicts remain to be resolved (Figures S3 and S4). These results corroborate the ruggedness of the energy landscape, which describes complexes of disordered regions. We illustrated through two examples that the fuzzy regions display distinct frustration patterns with different partners, rationalizing how residual frustration allows both specificity and versatility to be encoded. These observations can be exploited upon targeting disordered regions by small molecules. Our results indicate that binding small molecule drugs do not need to induce folding of disordered regions. Instead, specificity may originate from distinct frustration patterns of highly heterogeneous conformations via ’disordered binding’. Our work shows that specificity of the interaction is not solely encoded in a given contact pattern, but also by the way frustration is ameliorated. These observations highlight the importance of conflicting, suboptimal interaction in drug design for disordered regions.

The coupled folding and binding of disordered regions leads to suboptimal contacts, which thus allows binding to different partners. This is fully consistent with the original notion of local frustration in spin glasses and systems such as the triangular antiferromagnet where many structures compete as global minima.^23,33^ The high residual frustration explains why disordered regions are capable of manifesting several different binding modes.^25^ The frustration of interactions in disordered proteins and their bound complexes allows binding to be fuzzy by being suboptimal thereby enabling multifunctionality. Frustration and the ruggedness of the energy landscape thus enable functional versatility along with specificity.

## Supporting Information Available

Configurational and mutational frustration of disorder-to-order regions and context-dependent regions in their unbound (monomeric) forms; correlation between the configurational frustration indices in the monomeric and bound forms for DOR and CDR data set (PDF)
Pair distribution functions for the mutational frustration indices for DOR and CDR data set (XLSX)
