# Supplementary material for: Frustration in Fuzzy Protein Complexes Leads to Interaction Versatility
Source: J Phys Chem B. 2021 Mar 5;125(10):2513–20. doi: 10.1021/acs.jpcb.0c11068 (PMC8041309; doi:10.1021/acs.jpcb.0c11068)
Supplement: Supplementary file 1 — jp0c11068_si_001.pdf [file jp0c11068_si_001.pdf]

Frustration in fuzzy protein complexes leads to interaction versatility

Maria I. Freiberger[a], Peter G. Wolynes[b], Diego U. Ferreiro *[a] and Monika Fuxreiter *[d,c]

[a] Protein Physiology Lab, Departamento de Quimica Biologica, Facultad de Ciencias Exactas y Naturales, Universidad de Buenos Aires-CONICET-IQUIBICEN, Buenos Aires, Argentina

[b] Center for Theoretical Biological Physics, Rice University, Houston, USA

[c] Department of Biomedical Sciences, University of Padova, Padova, Italy

[d] Laboratory of Protein Dynamics, University of Debrecen, Hungary

E-mail: diegulise@gmail.com; monika.fuxreiter@unipd.it

Supporting Information

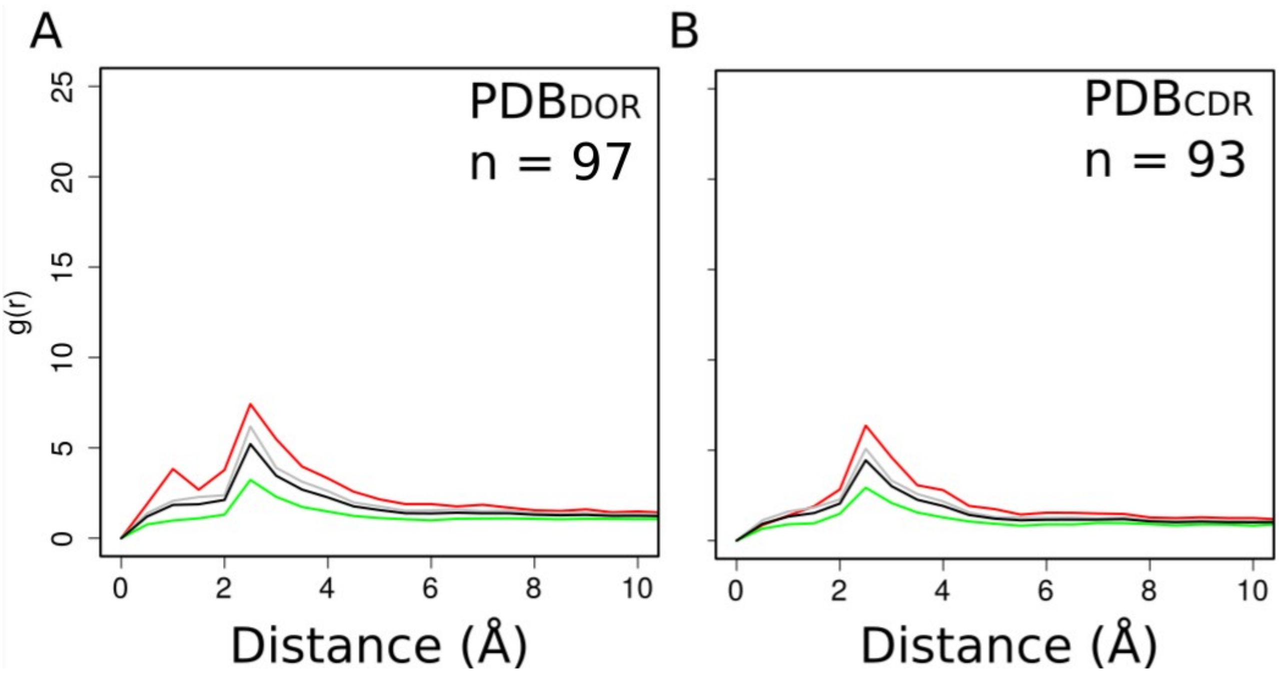

Figure S1. Mutational frustration of disordered regions in complexes generated by templated folding (A) andconditional folding (B). Pair distribution functions of the mutational frustration indices computed between the Cα of the annotated fuzzy residue in the bound form. Green: minimally frustrated contacts, red: highly frustrated, gray: neutral contacts, black: all contacts. g(r) plots were adjusted in their axis ranges to enhance visualizations, however in all cases g(r) values were normalized such that g(20)=1.

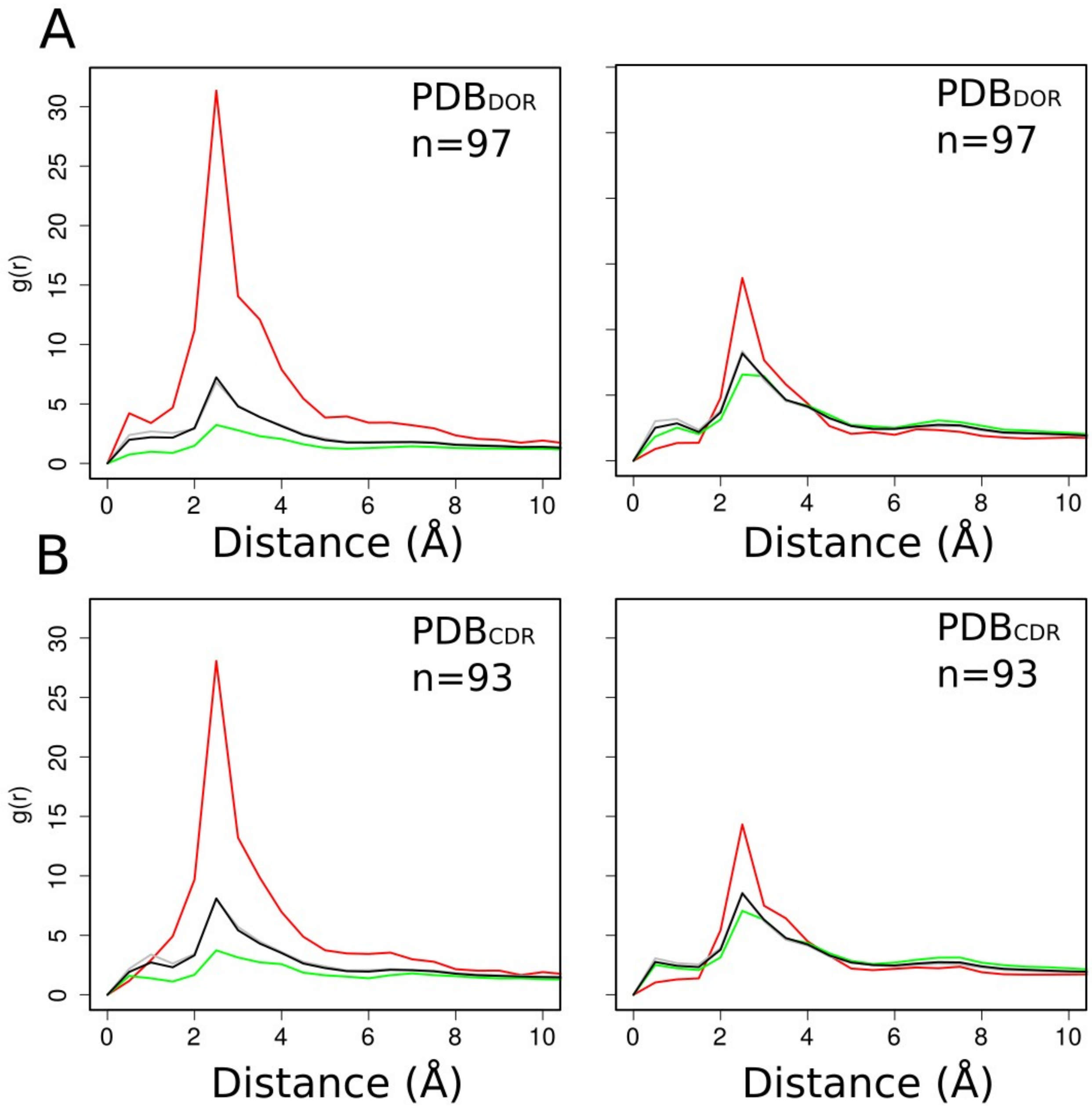

Figure S2. Configurational (left) and mutational (right) frustration of disorder-to-order regions (A) and context-dependent regions (B) in their unbound (monomeric) forms. (A) Disorder-to-order regions (DORs) undergo templated folding upon binding (B) Context-dependent regions conditionally fold upon binding to a specific set of partners. Pair distribution functions for single chains in the monomeric form were computed between the Cα's of the annotated fuzzy residues and the contacts in different classes. The configurational frustration index is shown on the left and mutational frustration index is shown on the right. Green: minimally frustrated contacts, red: highly frustrated, gray: neutral contacts, black: all contacts. g(r) plots were adjusted in their axis ranges to enhance visualizations, however in all cases g(r) values were normalized such that g(20)=1.

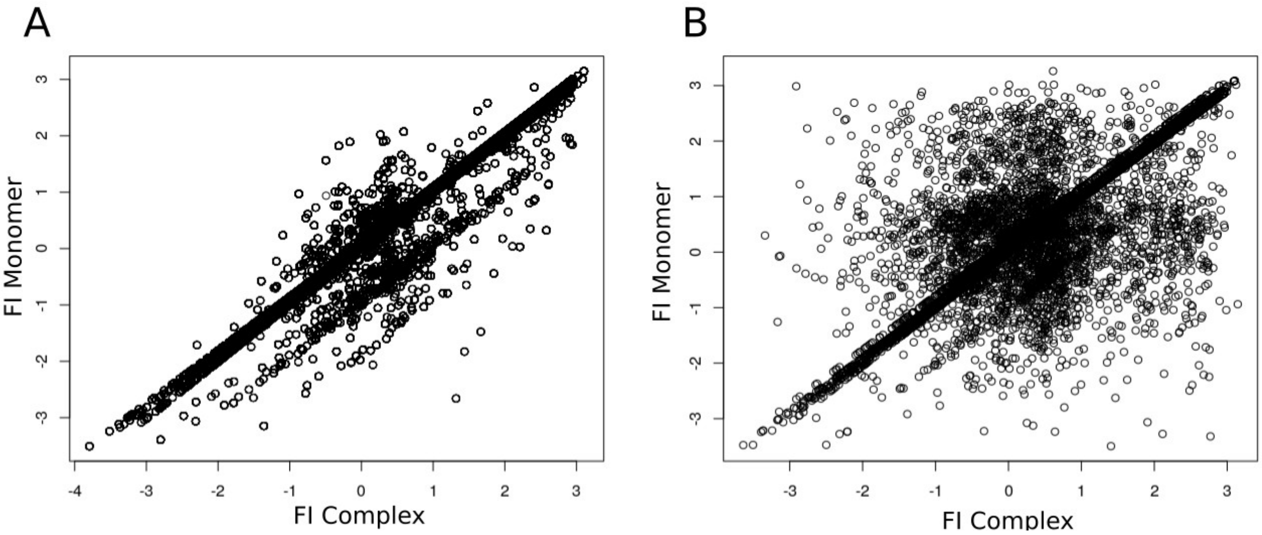

Figure S3. Templated folding reduces frustration of disordered regions as compared to their free forms. Correlation between the configurational frustration indices in the monomeric and bound forms are shown for contacts (A) involving residues of disorder-to-order (DOR) regions, (B) involving residues of structured regions.

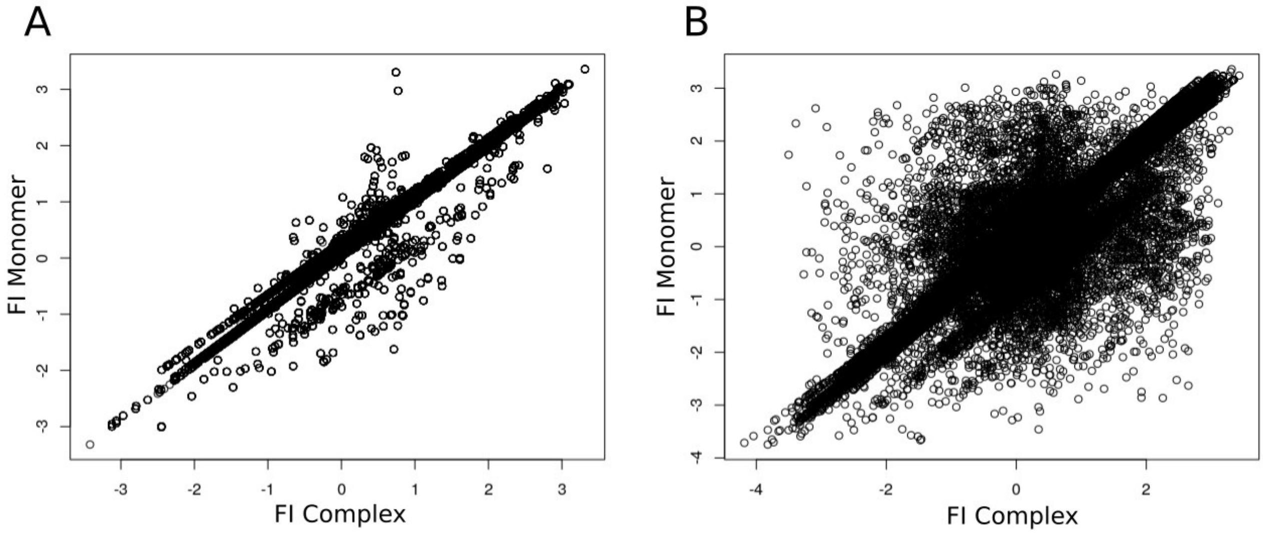

Figure S4. Conditional folding reduces frustration of disordered regions as compared to their free forms. Correlation between the configurational frustration indices in the monomeric and bound forms are shown for contacts (A) involving residues of context-dependent (CDR) regions, (B) involving residues of structured regions.

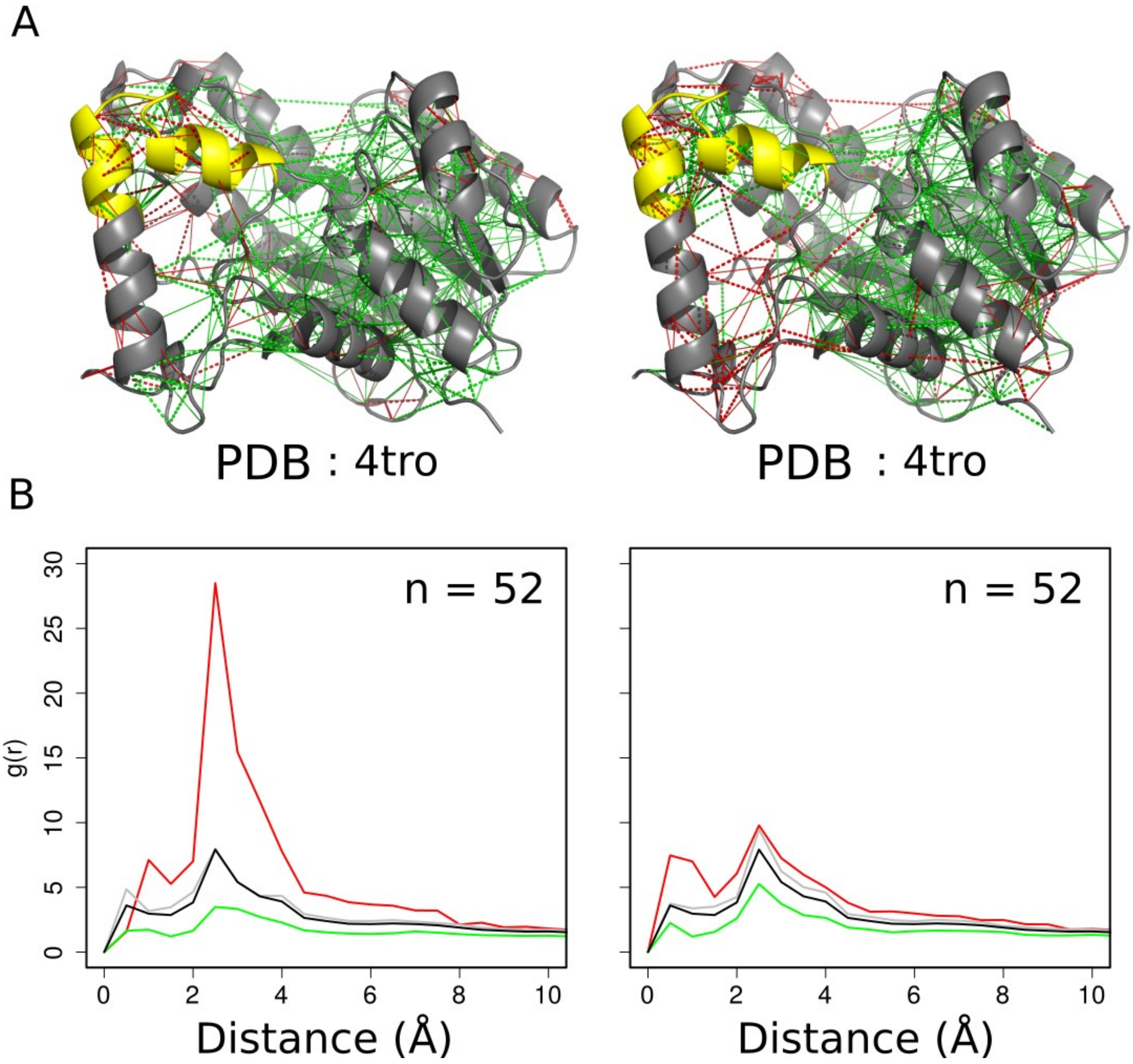

Figure S5. Frustration patterns of order-to-disorder regions. These regions are ordered in the unbound form and become disordered (unfold) upon binding. (A) Configurational (left) and mutational (right) frustration patterns are illustrated in the structure of enoyl-ACP reductase in complex with isoniazid (PDB:4tro). The backbones of the proteins are shown as gray cartoons, the fuzzy region is colored yellow (PBDID: 4tro, residues 197 - 215). The minimally frustrated contacts are depicted with green lines, highly frustrated interactions with red lines. Neutral interactions were omitted for clarity. (B) Pair distribution functions g(r) for configurational (left) and mutational (right) frustration indices, computed between the Cα of the annotated fuzzy residue and the rest of the protein. Green: minimally frustrated contacts, red: highly frustrated, gray: neutral contacts, black: all contacts. g(r) plots were adjusted in their axis ranges to enhance visualizations, however in all cases g(r) values were normalized such that g(20)=1.
